# Application of a novel numerical simulation to biochemical reaction systems

**DOI:** 10.3389/fcell.2024.1351974

**Published:** 2024-09-06

**Authors:** Takashi Sato

**Affiliations:** Digital Engineering Team, Production Tech. Lab, Research and Development Center, Zeon Corporation, Tokyo, Kanagawa, Japan

**Keywords:** biochemical reaction system, numerical simulation, algorithm, feedback, feedforward, Michaelis-Menten, allosteric, entropy

## Abstract

Recent advancements in omics and single-cell analysis highlight the necessity of numerical methods for managing the complexity of biological data. This paper introduces a simulation program for biochemical reaction systems based on the natural number simulation (NNS) method. This novel approach ensures the equitable treatment of all molecular entities, such as DNA, proteins, H_2_O, and hydrogen ions (H^+^), in biological systems. Central to NNS is its use of stoichiometric formulas, simplifying the modeling process and facilitating efficient and accurate simulations of diverse biochemical reactions. The advantage of this method is its ability to manage all molecules uniformly, ensuring a balanced representation in simulations. Detailed in Python, NNS is adept at simulating various reactions, ranging from water ionization to Michaelis–Menten kinetics and complex gene-based systems, making it an effective tool for scientific and engineering research.

## 1 Introduction

In recent years, single-cell and multi-omics data analysis has attracted attention in the field of molecular cell biology (Lim et al., 2023; Massimino et al., 2023; Baysoy et al., 2024). In particular, analysis using machine learning models, such as deep learning, has been active (Lin et al., 2022; Gunawan, et al., 2023; Wagle et al., 2024). However, understanding stoichiometric reactions is still considered essential to understand gene expression and protein enzyme functions in detail. This paper proposes a simulation program for biochemical reaction systems based on the natural number simulation (NNS) method. NNS offers a simplified approach for modeling biochemical reactions stoichiometrically and performing computations of the time course of the reactions. This method excels in its straightforward formulation process and is particularly suitable for complex biological systems (Blinov et al., 2004; Browning et al., 2022; Huizing et al., 2022) because of the absence of complicated reaction formulas using mathematical equations.

Various models have been considered to represent complex biological systems, including ordinary differential equations and Gillespie’s algorithm (Gillespie, 1977; Ullah and Wolkenhauer, 2011; Alon, 2019). For example, metabolic reaction systems continue to be vigorously analyzed in detail using differential equations (Khodayari and Maranas, 2016; Himeoka and Mitarai, 2022). Petri nets substantially contribute to analyzing biochemical reaction networks by integrating stochastic algorithms and/or non-parametric strategies. This progression has resulted in the emergence of specialized forms such as signaling Petri nets, large-scale metabolic models, and multilevel biological models using colored Petri nets, indicating their increasing utility in the analysis of complex systems (Ruths et al., 2008; Rohr, 2018; Brinkrolf et al., 2021; Shaikh et al., 2022; Liu et al., 2023).

However, ordinary differential equations require complex formulations of reaction rate equations. Stochastic simulations require detailed formulations of chemical reaction networks, which may limit their usefulness, particularly for large-scale simulations. Petri net-based simulations offer a comprehensive framework for formulating chemical reaction networks involving specific elements such as places, transitions, arcs, and markings. However, modeling actual biochemical reaction systems requires consideration of the definition of the number of tokens and their correspondence to the number of molecules. In addition, when introducing stochastic models, it is necessary to explicitly define the timing of firing.

The NNS method implemented in Python is very easy to model because the procedure for determining detailed time evolution is included in the computational algorithm. Requiring only the stoichiometric equation, its rate constants, and the initial number of molecules, the new algorithm, based on a probabilistic binomial distribution, immediately calculates the number of molecules after one calculation step for the elements in the model system. It is crucial to emphasize the necessity of precise rate constant determination to depict the system accurately. Nevertheless, the simplicity of the formulation has the advantage of allowing the optimization process to be easily performed.

Recent advancements in non-parametric analytical techniques also present an intriguing possibility for NNS (Ruths et al., 2008; Rohr, 2018). By meticulously setting the initial molecular counts, rate constants, and stoichiometric equations, an analysis comparable to those seen in studies of complex signaling networks may be achieved. This approach could potentially enhance the applicability and accuracy of NNS for modeling intricate biochemical systems.

Contrary to traditional methods, such as ordinary differential equations and Gillespie’s algorithm, which might not optimally represent complex biological systems (Gillespie, 1977; Alon, 2019), NNS provides a more precise simulation even for a small number of molecules in DNA-related reactions. It uses stoichiometric equations with rate constants for specific and selective processes such as transcription and translation (Watson et al., 2014). Moreover, the binomial distribution in NNS facilitates calculating informational and entropic metrics, including Shannon’s entropy (Shannon, 1948; Adriaans and van Benthem, 2008). Examples of these calculations are discussed later in the paper.

## 2 Methods

Our method calculates time-developing stoichiometric reactions using a binomial algorithm. Consider the general reaction as Equation 1:

$$q_{1}X_{1}+q_{2}X_{2}+\cdots +q_{s}X_{s}\overset{k}{\to }r_{1}Y_{1}+r_{2}Y_{2}+\cdots +r_{t}Y_{t}$$ (1)

*X*_*1*_, *X*_*2*_, ⋯ , *X*_*s*_ denote molecular elements before the reaction while *Y*_*1*_, *Y*_*2*_, ⋯ , *Y*_*t*_ represent those after the reaction. The coefficients q_i_ and r_i_ are reaction orders. The subscripts “*s*” and “*t*” are natural numbers, with no limitation on their sizes. The rate constant k implies the probability of forming *Y*_*1*_, *Y*_*2*_, ⋯ , *Y*_*t*_. In the NNS approach, reaction orders are consistently natural numbers because they necessitate dealing with element counts as natural numbers at all times. Our methodology accommodates stoichiometric reactions without limiting specific chemical formulas, thereby ensuring the conservation of the atom number.

Initial element counts are necessary for computing the time evolution. *X*_*i*_ *_n* and *Y*_*i*_ *_n* are defined as the elemental number of *X*_*i*_ and *Y*_*i*_; then, the following calculation in Equations 2, 3 with Equations 4, 5 provide the decrement and increment quantities of the elements.

$$∆X_{i}\_n=-q_{i}\times R_{binomial}\left(n,p\right)$$ (2)

$$∆Y_{i}\_n=r_{i}\times R_{binomial}\left(n,p\right)$$ (3)

$$n={\mathrm{int}\left(\frac{X_{i}\_n}{q_{i}}\right)}_{\min}$$ (4)

$$p=\prod_{i\neq \min}\frac{k∙\left(\frac{X_{i}\_n}{q_{i}∙N}\right)}{1+k∙\left(\frac{X_{i}\_n}{q_{i}∙N}\right)}$$ (5)

*R*_*binomial*_(*n*, *p*) generates random numbers following the binomial distribution, returning natural numbers (Rohr, 2018). In this function, *n* denotes a trial number, each of which can be either a success or a failure (binary outcomes). The parameter *p* specifies the probability of success in each trial. The function provides the total number of successes in n trials. NumPy’s random binomial (n, p) achieves this. The function returns one successful natural number under *n*, including zero. Stochastic simulation often uses these discrete numbers (Székely and Burrage, 2014; Gholami and Ilie, 2021). The trial number *n* is represented with the minimum integer value among *X*_*1*_ *_n*/*q*_*1*_, *X*_*2*_ *_n*/*q*_*2*_, *X*_*3*_ *_n*/*q*_*3*_, so on. The probability p is derived based on the rate constant k, the reaction order *q*_*i*_, a normalization parameter N, and *X*_*i*_ *_n*, except for $\frac{k∙\left(\frac{X_{i}\_n}{q_{i}∙N}\right)}{1+k∙\left(\frac{X_{i}\_n}{q_{i}∙N}\right)}$ with $\frac{X_{i}\_n}{q_{i}}$ selected in Equation 4. In a calculation step, *X*_*i*_ *_n* becomes *X*_*i*_ *_n* + *ΔX*_*i*_ *_n* and *Y*_*i*_ *_n* becomes *Y*_*i*_ *_n* + *ΔY*_*i*_ *_n*. The rate constant k in NNS is semantically the same as the rate constant that appears in the stoichiometric equation defined by concentration. However, k in this study is defined as a constant that affects the stochastic results defined in Equation 5. The rate constant k, the number of elements, and the normalization constant N determine the probability p, as indicated by Equation 5. The k value influences the probability variation between 0 and 1 and the reaction rate, but it is not the same as the conventional rate constant.

Equations 4, 5 have some minor formulas. One is in the case of a one-element reaction as Equation 6:

$$\text{aX}\to r_{1}Y_{1}+r_{2}Y_{2}+\cdots +r_{t}Y_{t}\text{ with }a\text{ rate constant }k$$ (6)

*X* is a molecular element, and *a* is an order. The decrement *ΔX_n* and increment *ΔY*_*i*_ *_n* are derived from the following formulas as Equations 7, 8 with Equations 9, 10:

$$∆X\_n=-a\times R_{binomial}\left(n,p\right)$$ (7)

$$∆Y_{i}\_n=r_{i}\times R_{binomial}\left(n,p\right)$$ (8)

$$n=\mathrm{int}\left(\frac{X\_n}{a}\right)$$ (9)

$$p=\frac{k}{1+k}$$ (10)

where *X_n* is the number of the element *X*.

Another exceptional reaction type is as Equation 11:

$$\text{aX }\to 0\;\left(\text{zero}\right),\text{with }a\text{ rate constant }k$$ (11)

This expression indicates that element X is decomposed and disappears, so Equation 3 does not work. Thus, only X decreases with Equation 7 with Equation 9, 10.

Another type of reaction is defined as a linear decreasing and increasing reaction for one element. One needs these reactions for the mathematical formulation of linear changes via molecular addition and subtraction. The linear decreasing case is defined similarly to Equation 11 as Equation 12:

$$\text{aX }\to 0\;\left(\text{zero}\right)\text{ by linear},\text{with }a\text{ rate constant }k$$ (12)

Moreover, the increment of X is determined by the following Equation 13:

$$∆X\_n=-a\times R_{binomial}\left(1,p\right)$$ (13)

where *p* is the same as in Equation 10.

Subsequently, the linear increasing case is defined by the following reaction as Equation 14:

$$0\;\left(zero\right)\to \;bY\text{ by linear},\text{with }a\text{ rate constant }k$$ (14)

The following Equation 15 also determines the increment of Y:

$$∆Y\_n=b\times R_{binomial}\left(1,p\right)$$ (15)

where *p* is the same as in Equation 10.

A biological system has numerous incredible reactions (Nelson and Cox, 2021). One can easily define each reaction if one knows the elements before and after the reaction, their orders, and rate constants.

Executing an input file in Spyder, an Integrated Development Environment requires setting “fName = “inp_file.txt”” in the main program (i.e., binomial_v016.py) and then utilizing the “Run” command. For command line execution, the command “$ python binomial_v016.py inp_file.txt” yields similar outcomes to Spyder’s run. Therefore, it is essential to position the input file in the program’s directory or one level above it.

This file needs to define calculation time, elements, reactions, and plots (Table 1). Currently, model systems are defined in text files. Currently, a Python program is available on GitHub that converts xml files that conform to Systems Biology Markup Language into text files for NNS. Users can perform calculations via NNS by setting the appropriate initial number of elements and rate constants.

**TABLE 1:** Standard input file style. The *Time, *Element, *Reaction, and *Plot are commands for calculation in an input file.

*Time					
Start time	End time	Console output interval time	Plot output interval time	CSV-file output interval time	Time unit
*Element					
Element name	Initial number	Color for *Plot (optional)	Marker type (optional)		
*Reaction	Normalization parameter				
Type Name	Identification name	Before elements Order, element name	Rate constant (k)	After elements Order, element name	
*Plot	Plot type				
Element names					

The *Reaction statement has numerous definitions (Supplementary Tables S1, S2), including the above formulas used to calculate reaction increments.

**FIGURE 1:**
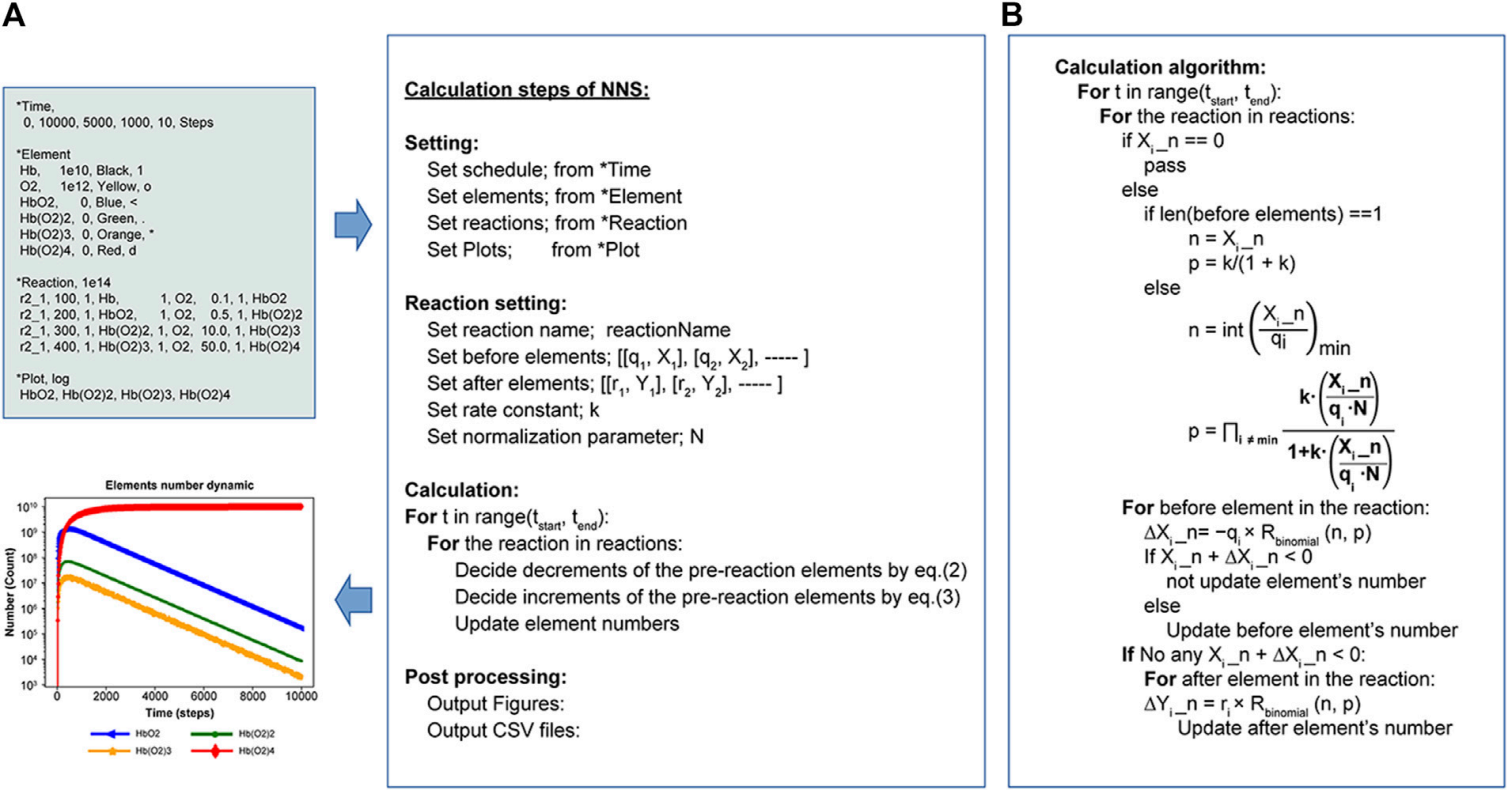
Calculation flow and algorithm of NNS. **(A)** Workflow executed by Python programs. The upper left shows an input file (Supplementary Figure S2A for an example), and the lower left depicts the resulting graph (Figure 5 for an example). **(B)** Detailed NNS calculation algorithm of the calculation in the Calculation steps of NNS: **(A)**.

The *ElementInOut statement implemented definition statements to express the inflow and outflow of molecules in and out of the system (Supplementary Table S3). The author developed the Python program used for this algorithm, and it is available under the MIT license on GitHub at https://github.com/taka-b/binomial/tree/binomial_v016_01 (Sato, 2023). This algorithm is publicly accessible and can be used by anyone without any charge. Figure 1A shows the calculation for schematic flow, while Figure 1B depicts the detailed calculation algorithm. The simplicity of the proposed procedure is demonstrated through six examples using their corresponding input files.

## 3 Results

Some example calculations are shown below. Note that the user only needs to prepare a text file for the calculations to obtain the results. The important point is to carefully formulate the stoichiometric equation and give appropriate rate constants.

The following results are obtained for calculations based on the text file shown in each figure. The start and end times are defined by *Time; for example, zero, 10,000, displays the results of calculations for every step from zero to 10,000. If you want to compare these results with actual experimental results, you will need units and rate constants that correspond to the experimental results. Here, however, the units of calculation are denoted in Steps, and figures show changes in the number of elements.

### 3.1 Simple reaction model

Consider a simple reaction with rate constant *k*_*on*_ as Equation 16:

$$A+B\;\to C,\text{with rate constant }k_{on}$$ (16)

where *A* and *B* represent protein molecules that irreversibly bind to form *C*.

**FIGURE 2:**
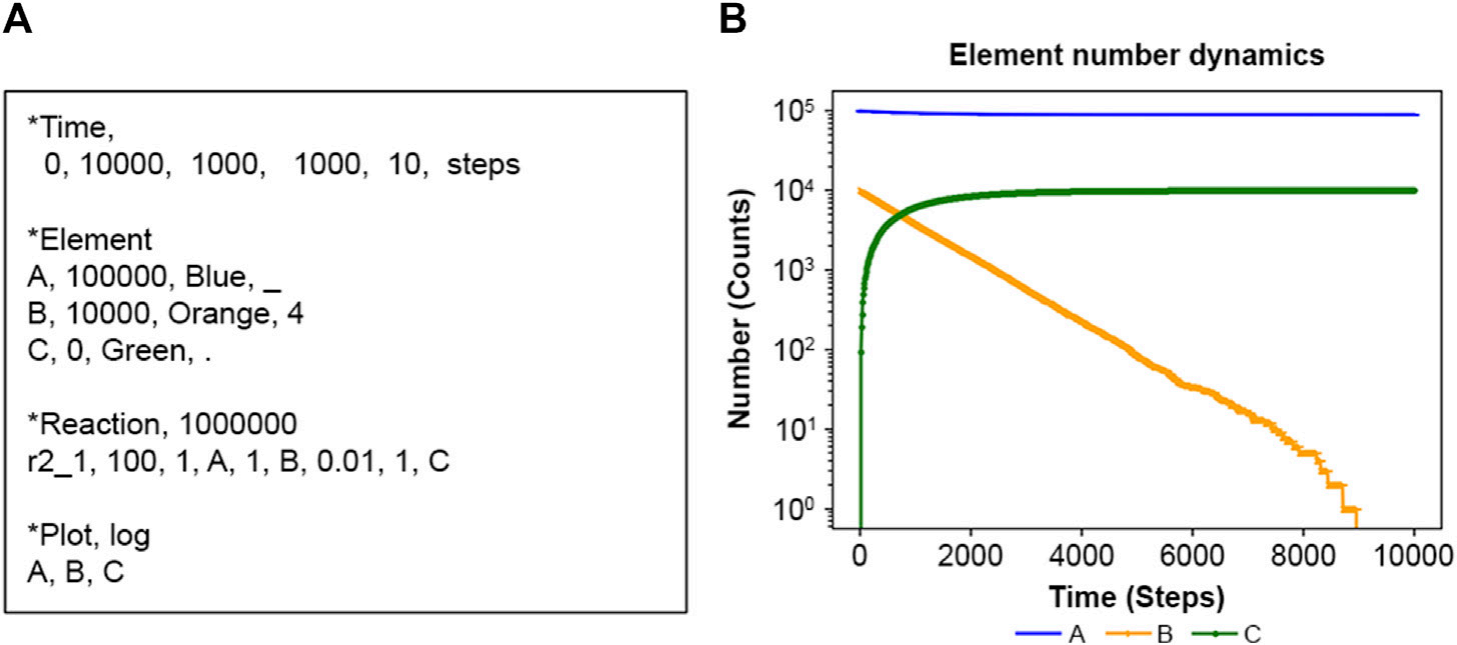
**(A)** Input text and **(B)** calculation result of **(A)**.

Figure 2A is an input file (details in Table 1) defining one reaction r2_1_100 (details in Supplementary Table S1), where the calculation spans time zero to 10,000 in dimensionless units. In the actual calculation, each increase or decrease in the number of elements is calculated according to the above algorithm for each increase or decrease. The user must assign the appropriate time unit for a particular system. Here, for the sake of schematic calculation, the unit of time is used as the calculation unit Steps. NNS uses natural numbers, and as shown in Figure 2B, the *y*-axis represents the number of elements, guaranteeing an accurate number of elements.

In contrast, Supplementary Figure S1 introduces the inverse reaction r1_2_200 as Equation 17.

$$C\;\to A+B,\text{with rate constant }k_{\text{off}}$$ (17)

The rate constant k_off_ differs from k_on_ in Equation 16 due to the thermodynamic principle in the system (Craig et al., 2021). Supplementary Figure S1 shows the equilibrium numbers of A, B, and C.

### 3.2 Water ionization

**FIGURE 3:**
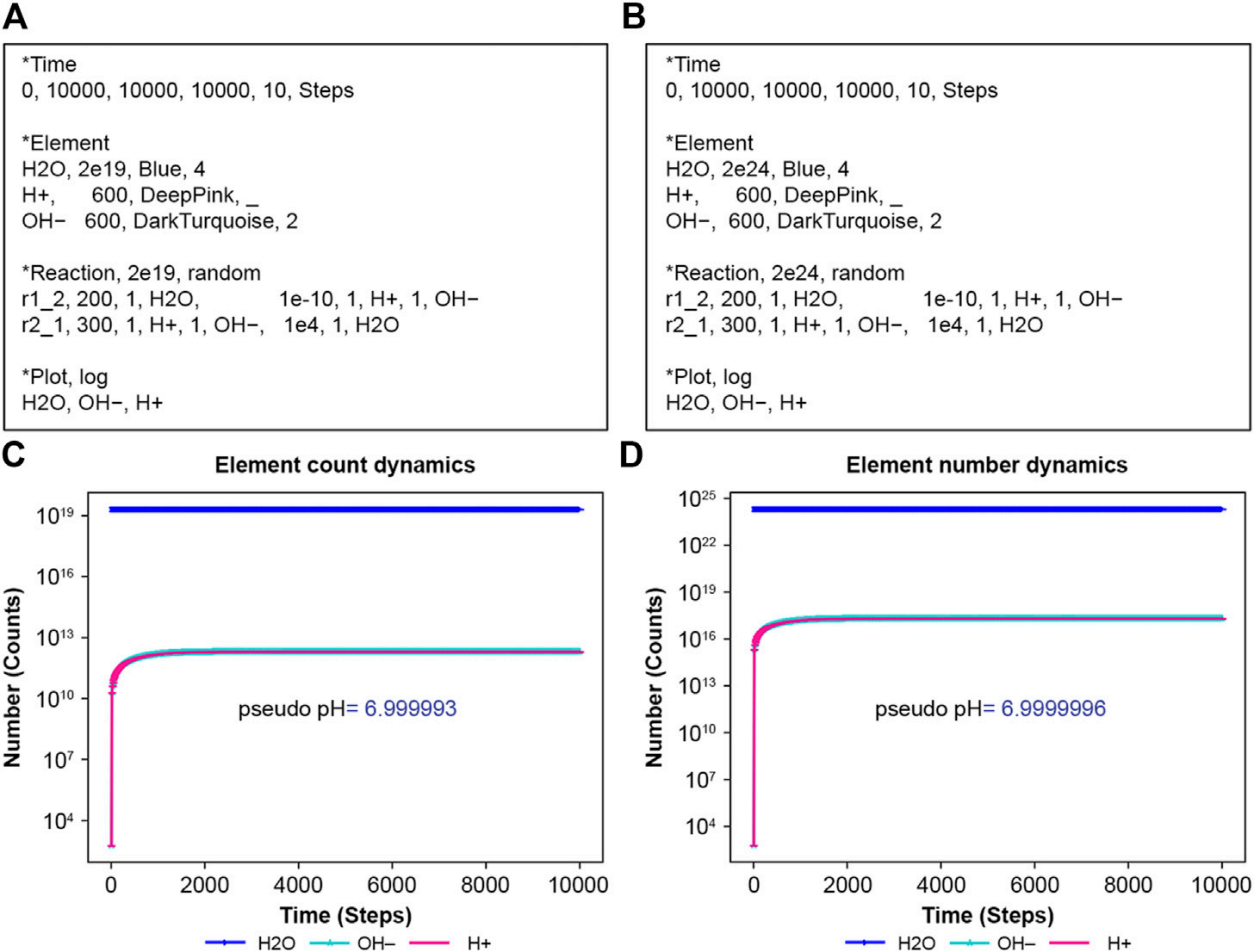
Ionization of water. H_2_O means water, H_2_O; H+ hydrogen ion H^+^; and OH–hydroxide ion, OH^−^. **(A)** Input text with N = 2e19 and **(B)** with N = 2e24. **(C)** The calculation result of **(A,D)** that of **(B)**.

NNS can perform water ionization. Water, H_2_O, usually dissociates slightly into proton ions (H^+^) and hydroxyl ions (OH^−^), representing a dynamical equilibrium between dissociation and reassociation. Figures 3A, B are input files in this scenario. Suitable rate constants show this equilibrium in Figures 3C, D. Notably, NNS accounts for system size through normalization parameters. Although the input files present different initial water molecule counts, their rate constants remain consistent. These normalization parameters adjust the system size by changing the success probability, p, in Equation 5. If a pseudo pH is defined as Equation 18:

$$pseudo\;pH=-\log_{10}\left(\frac{number\;of\;H+}{number\;of\;H2O}\right)$$ (18)

The results from Equation 18 using Figures 3B, D simulating data have almost the same values, 6.999993 and 6.9999996, respectively. The exact rate constants for the dissociation of water and recombination of proton ions (H^+^) and hydroxyl ions (OH^−^) remain unknown. Therefore, the horizontal axis in Figures 3C, D is labeled in Steps, the unit of calculation. If the exact values of these rate constants are known, the appropriate unit of time should be determined accordingly.

### 3.3 Michaelis–Menten model

**FIGURE 4:**
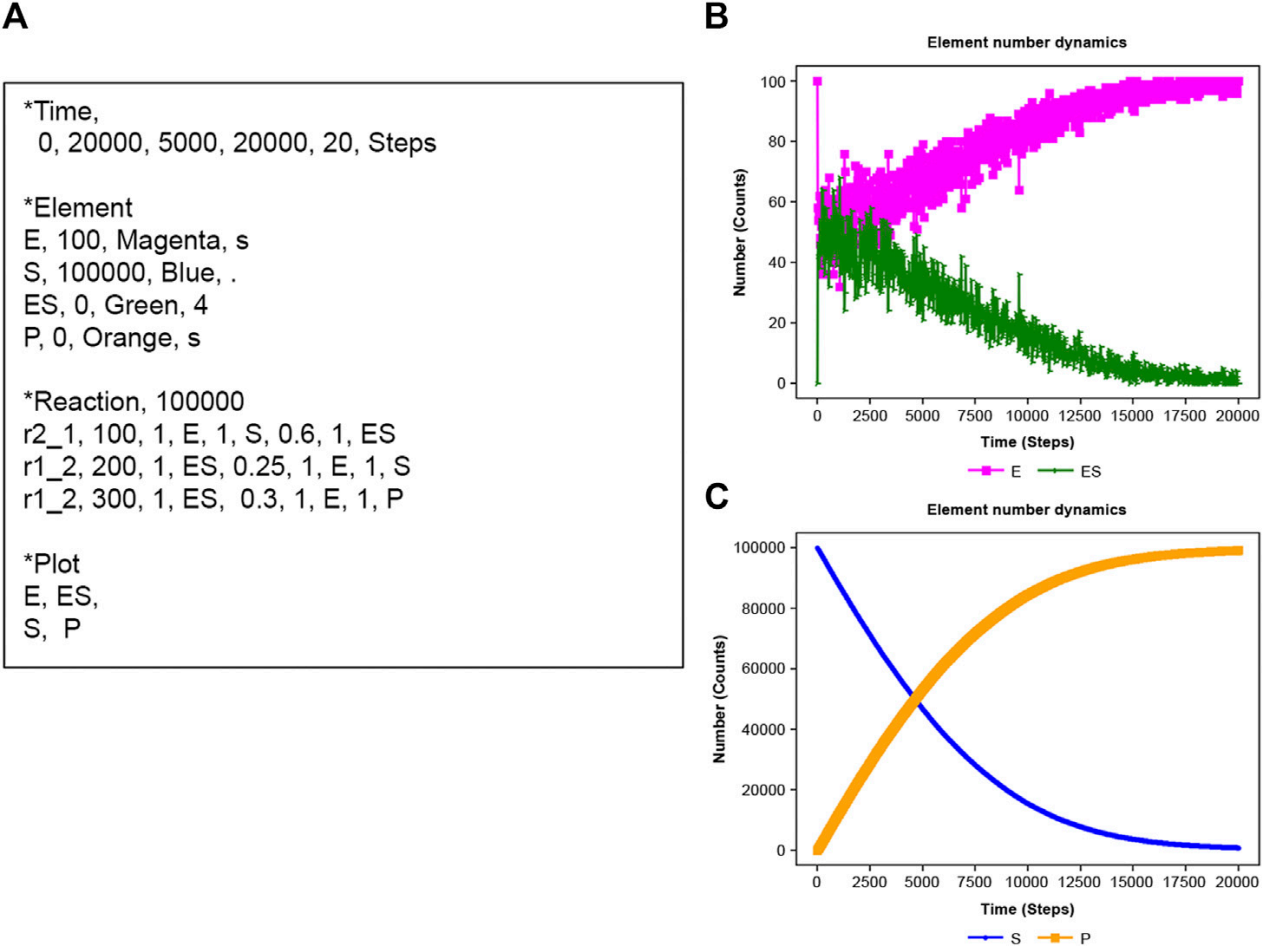
Michaelis–Menten kinetics. **(A)** Input text. **(B)** Results of E (enzyme) and ES enzyme-substrate complex). **(C)** Results of S (substrate) and P (product).

Michaelis–Menten kinetics (Gilbert et al., 2006) is applied using NNS. In Figure 4A, E is an enzyme, S is the substrate, ES is the enzyme-substrate complex, and P is the product (Craig et al., 2021). The corresponding scheme is as Equation 19:

S+E ⇄ES →E+P (19)

NNS defined three reactions in the input file. The total E number (E_n + ES_n) is constant, though E and ES fluctuate in time series (Figure 4B). Furthermore, S decreases with increasing P (Figure 4C).

### 3.4 Monod–Wyman–Changeux allosteric model

**FIGURE 5:**
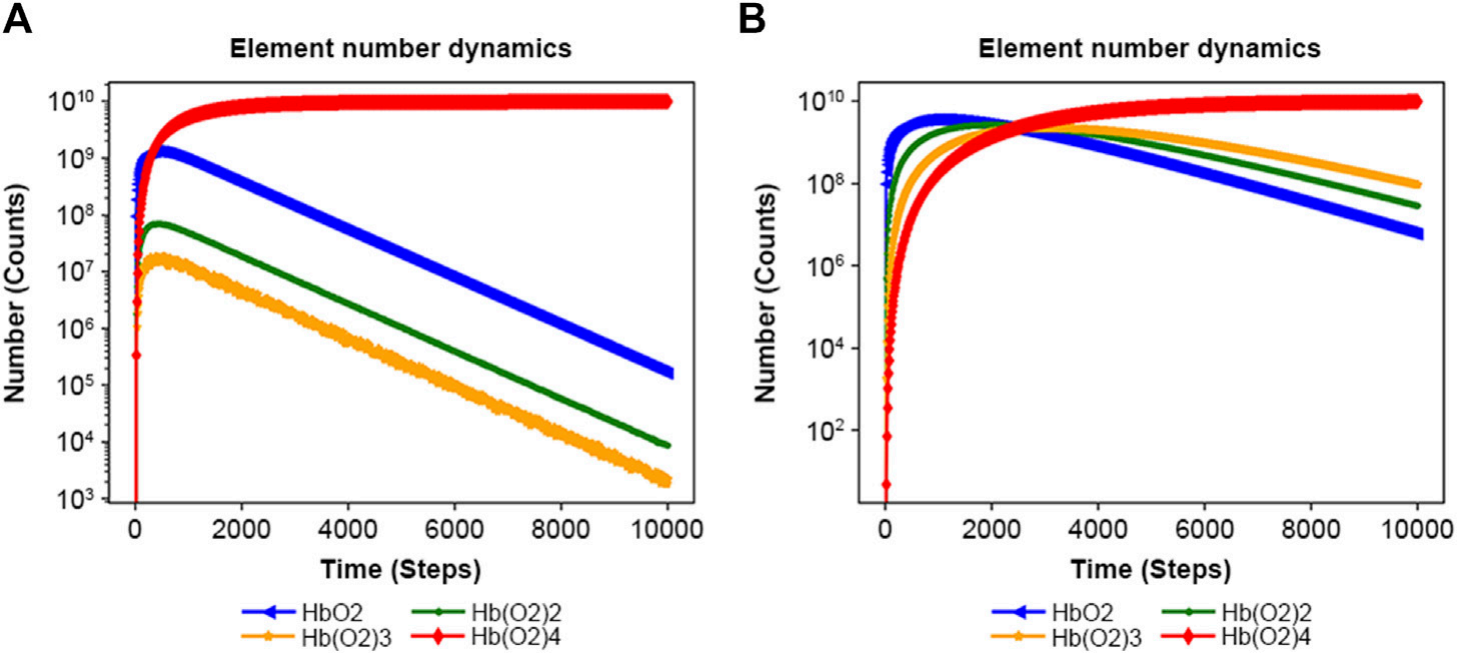
Results of the Monod–Wyman–Changeux allosteric model. **(A)** Result of Supplementary Figure S2A with rate constants: 0.1, 0.5, 10, and 50. **(B)** Result of Supplementary Figure S2B, with rate constants: 0.1, 0.1, 0.1, and 0.1.

NNS can manage allosteric models (Goodey and Benkovic, 2008; Machado et al., 2015; Wodak et al., 2019). The allosteric transition of hemoglobin is one of the most interesting behaviors because of molecular adaptation in vertebrates (Eaton, 2022). Supplementary Figure S2A features an input file for the binding of oxygen to hemoglobin at different rate constants, 0.1, 0.5, 10, and 50. However, Supplementary Figure S2B uses a constant rate of 0.1. Figure 5A illustrates the allosteric effect; its increasing rate of oxyhemoglobin (Hb(O_2_)_4_) binding was higher than that observed in the no allosteric effect model of Figure 5B initially (Supplementary Figure S3A). The ratios of Hb(O_2_)_4_ to other complexes are also consistently higher than those observed in the non-allosteric model (Supplementary Figure S3B).

### 3.5 Feedback loop model

**FIGURE 6:**
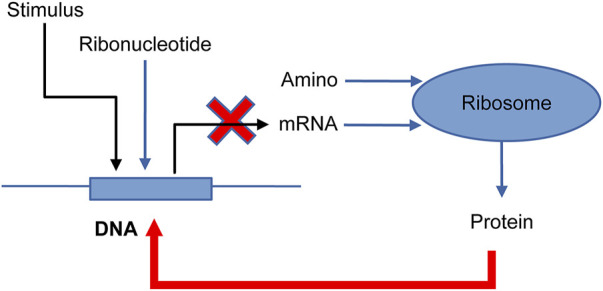
Feedback system model for one-gene DNA (Alon, 2019). Stimulus: protein molecules bind DNA; DNA: one gene; Ribonucleotide: some ribonucleotides; mRNA: messenger-RNA; Amino: some amino acids; Ribosome: ribosome for translation; and Protein: a protein made by the ribosome. DNA is transcribed into mRNA, which is then translated into Protein. The protein binds to DNA to deactivate and is modified into DNA_d (deactivate). (Reproduced from Alon, 2019, with permission from Chapman and Hall/CRC).

**FIGURE 7:**
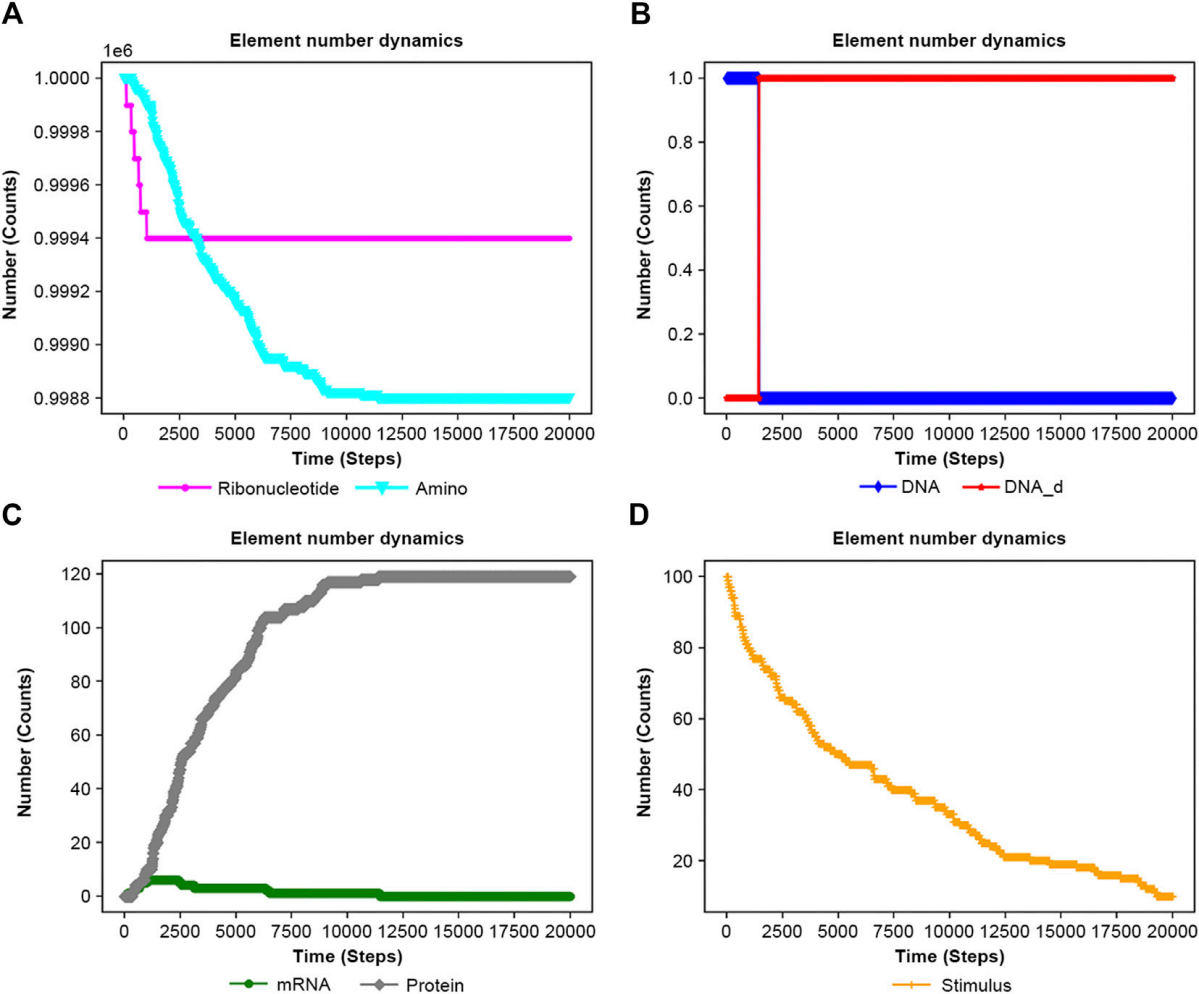
Results of the feedback system of the input file are in Supplementary Figure S4. **(A)** RNA and Amino. **(B)** DNA and DNA_d. **(C)** mRNA and Protein. **(D)** Stimulus.

NNS supports the models of feedback loops in biological systems, which are common regulatory mechanisms (Ferrazzi et al., 2011; Alon, 2019). Figure 6 depicts a feedback loop where a stimulus activates DNA, but the resultant protein then deactivates DNA into DNA_d (deactivated DNA). Supplementary Figure S4’s input file models ribonucleotide (Ribonucleotide) and amino acid (Amino) as singular types. The advantage of NNS is its ability to represent individual DNA molecules and deliver their specific properties. In Figure 7 the DNA is inactivated by the protein, mRNA production is stopped, and protein production is suppressed.

Figure 7 and Supplementary Figure S5 exhibit contrasting results with and without feedback, respectively, highlighting the protein’s role in limiting DNA activation in feedback systems.

### 3.6 Feed-forward loop in a biological system

**FIGURE 8:**
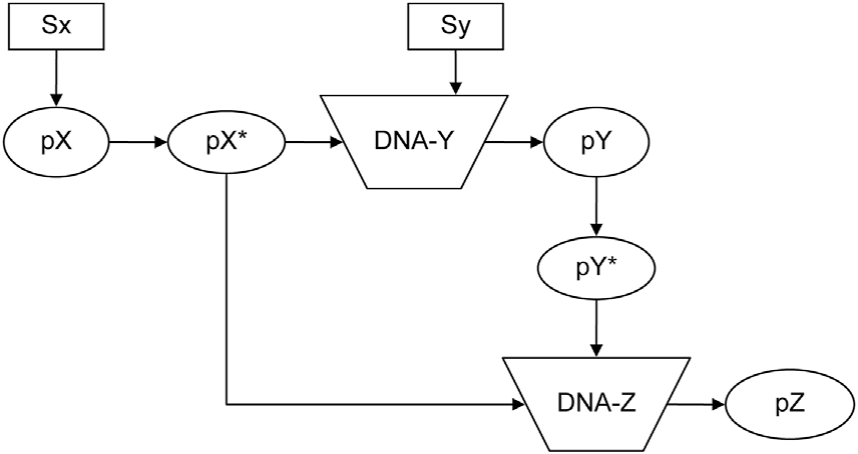
Feed-forward system (Alon, 2019). Sx and Sy: Stimuli; pX, pY, and pZ: proteins; pX* and pY*: activated proteins of pX and pY; DNA-Y and DNA-Z: DNA for each protein pY and pZ. (Reproduced from Alon, 2019, with permission from Chapman and Hall/CRC).

**FIGURE 9:**
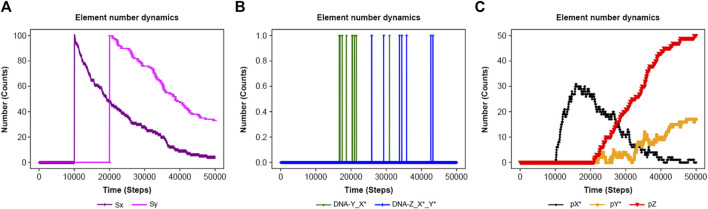
Results of the feed-forward system in Supplementary Figure S6 until time = 50,000 steps. Sx and Sy are added at 10,000 and 20,000 steps, respectively. **(A)** Sx and Sy. **(B)** DNA-Y_X* and DNA-Z_X*_Y*. **(C)** pX*, pY*, and pZ.

The feed-forward loop is a prevalent biological system (Kremling et al., 2008; Macía et al., 2009; Le and Kwon, 2011). Figure 8 depicts a schematic reaction process (Alon, 2019). Stimuli Sx and Sy ultimately promote protein pZ production. The system encompasses 10 elements, two input elements and six reactions, whose detailed functions are shown in the input file of Supplementary Figure S6. Using *elementInOut (Supplementary Table S3) and shifting the inflow timing of Sx and Sy as shown in Supplementary Figure S6 affects the behavior of the protein, as shown in Figure 9: Sy is inputted later than Sx, the production of Pz is delayed. On the other hand, in Supplementary Figures S7, S8, Sx and Sy are inputted simultaneously, there is no significant delay in Pz. The flexibility of NNS proves to be a valuable tool for modeling such a system.

## 4 Discussion

Entropy and information are concepts related to information theory (Baez and Pollard, 2016), and stochastic processes with probability parameters can enrich our understanding of reaction systems. In our approach, parameters *n* and *p* are determined via reaction conditions and are described in the Methods section. The information on reaction is defined as Equation 20:

$$I\_reaction\left(t,X_{t};n_{t},p_{t}\right)=-\log_{2}\left(binomial\left(X_{t};n_{t},p_{t}\right)\right)$$ (20)

where binomial ( X;n,p ) represents a binomial distribution function with a stochastic variable *X* (Successes) determined using *n* and *p* for each reaction step *t* (Chanda et al., 2020), as illustrated in Supplementary Figure S9.

The I_reaction provides insight into the reaction dynamics, and its value increases as reactions proceed.

Drawing from the Shannon entropy (Ben-Naim, 2012), the reaction entropy (RE) can be defined as Equation 21:

$$RE\left(t;n_{t},p_{t}\right)=-\sum_{X_{t}=0}^{n_{t}}binomial\left(X_{t};n_{t},p_{t}\right)\log_{2}\left(binomial\left(X_{t};n_{t},p_{t}\right)\right)$$ (21)

The RE is illustrated in Supplementary Figure S10 as a function of *n* and *p*, and it captures the inherent randomness in reaction and activity of reactions (Baez and Pollard, 2016; Roach, 2020; Uda, 2020).

Using a DNA-type reaction example from Figure 7 and Supplementary Figure S4, the accumulated information on the reaction is calculated using Equation 22.

$$IR_{accumulate}\left(t,X_{t};n_{t},p_{t}\right)=-\sum_{t_{k}=0}^{t}\log_{2}\left(binomial\left(X_{t_{k}};n_{t_{k}},p_{t_{k}}\right)\right)$$ (22)

**FIGURE 10:**
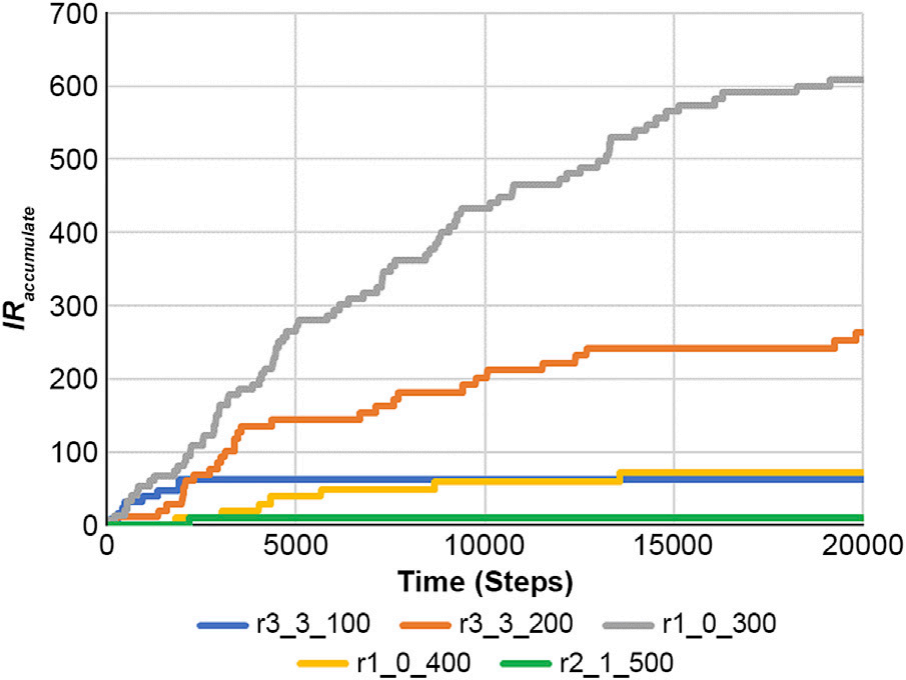
Accumulated information on the reaction was obtained using Equation 22 in the case of a feedback loop shown in Figure 7 and Supplementary Figure S3.

**FIGURE 11:**
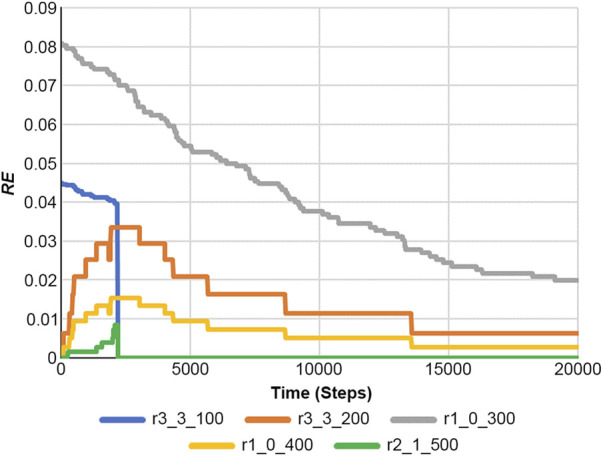
Reaction entropies of Equation 21 at any time in the case of a feedback loop of Figure 7 and Supplementary Figure S3.

Figure 10 shows the results of Equation 22, and Figure 11 presents the RE values. Here, the RE values represent the results obtained from Equation 21 using Figure 7 and Supplementary Figure S4. Information on reaction and RE offers fundamental tools for dissecting reaction properties and understanding the complexity of biochemical systems.

## Figures and Tables

**Table:** 

**X**_**i**_ **_n**	Elemental number of X_i_
**Y**_**i**_ **_n**	Elemental number of Y_i_
**ΔX**_**i**_ **_n**	Increment of elemental number of X_i_
**ΔY**_**i**_ **_n**	Increment of elemental number of Y_i_
**Hb(O**_**2**_ **)**_**x**_	Hemoglobin Bound by x Number of Oxygen Molecules
**DNA_d**	Deactivated DNA
**Amino**	Amino Acid Molecules

## Data Availability

The datasets presented in this study can be found in online repositories. The names of the repository/repositories and accession number(s) can be found in the article/Supplementary Material.
