# Supplementary material for: Application of a novel numerical simulation to biochemical reaction systems
Source: Front Cell Dev Biol. 2024 Sep 6;12:1351974. doi: 10.3389/fcell.2024.1351974 (PMC11412882; doi:10.3389/fcell.2024.1351974)
Supplement: Supplementary file 1 [file DataSheet1.docx]

*Supplementary Materials*

Application of a Novel Numerical Simulation to Biochemical Reaction Systems

Takashi Sato

* Correspondence: Corresponding Author: [sato@zeon.co.jp](mailto:sato@zeon.co.jp)

# Supplementary Figures and Tables

There are several supplementary tables (Table S1–Table S3) and figures (Figure S1–Figure S10).

## Supplementary Tables

**Supplementary Table S1**. Standard reaction types and definition styles for natural number simulation (NNS). In definition, the reaction name is r1_1_name, etc., for example.

| **Type name** | **Identification name** | **Before element:**  **Order and element name** | **Rate constant** | **After element:**  **Order and element name** | **Equation** |
| --- | --- | --- | --- | --- | --- |
| r1_0 | name | q_1_, X_1_, | k, | None | eq(9) |
| r1_1 | name | q_1_, X_1_, | k, | r_1_, Y_1_ | eq(6) |
| r1_2 | name | q_1_, X_1_, | k, | r_1_, Y_1_, r_2_, Y_2_ | eq(1) |
| r1_3 | name | q_1_, X_1_, | k, | r_1_, Y_1_, r_2_, Y_2_, r_3_, Y_3_ | eq(1) |
| r2_1 | name | q_1_, X_1_, q_2_, X_2_, | k, | r_1_, Y_1_ | eq(1) |
| r2_2 | name | q_1_, X_1_, q_2_, X_2_, | k, | r_1_, Y_1_, r_2_, Y_2_ | eq(1) |
| r2_3 | name | q_1_, X_1_, q_2_, X_2_, | k, | r_1_, Y_1_, r_2_, Y_2_, r_3_, Y_3_ | eq(1) |
| r3_1 | name | q_1_, X_1_, q_2_, X_2_, q_3_, X_3_ | k, | r_1_, Y_1_ | eq(1) |
| r3_2 | name | q_1_, X_1_, q_2_, X_2_, q_3_, X_3_ | k, | r_1_, Y_1_, r_2_, Y_2_ | eq(1) |
| r3_3 | name | q_1_, X_1_, q_2_, X_2_, q_3_, X_3_ | k, | r_1_, Y_1_, r_2_, Y_2_, r_3_, Y_3_ | eq(1) |
| r1_- | name | q_1_, X_1_, | k, | 0, 0 | eq(10) |
| r1_+ | name | 0, 0 | k, | r_1_, Y_1_ | eq(12) |

**Supplementary Table S2**. rM-reaction definition style for natural number simulation (NNS)

| **Type name** | **Identification name** | **Before element:**  **Order, element name** | **Equation** |
| --- | --- | --- | --- |
|  |  | rate constant |  |
|  |  | After element:  Order, element name |  |
| rM | name | q_1_, X_1_, q_2_, X_2_, q_3_, X_3_, - - - | eq(1) |
|  |  | k, |  |
|  |  | r_1_, Y_1_, r_2_, Y_2_, r_3_, Y_3_, - - - |  |

**Supplementary Table S3**. Additional statements on the input file, *ElementInOut

| ***ElementInOut** | |  |  |  |  |  |  |  |  |  |
| --- | --- | --- | --- | --- | --- | --- | --- | --- | --- | --- |
| Element name | Initial number | Color for *Plot | Marker type | Element type  (optional) | type-0 | A  time | Amount | A time | Amount | And so on |
| Element name | Initial number | Color for *Plot | Marker type | Element type  (optional) | type-1 | Interval  time | Amount |  |  |  |

## Supplementary Figures


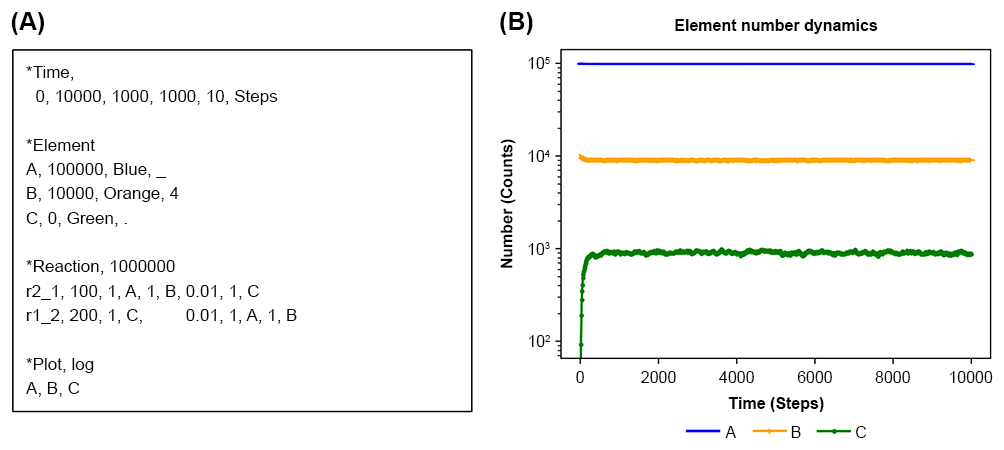


**Supplementary Figure S1.** **(A)** Input text. **(B)** Calculation results of (**A**).


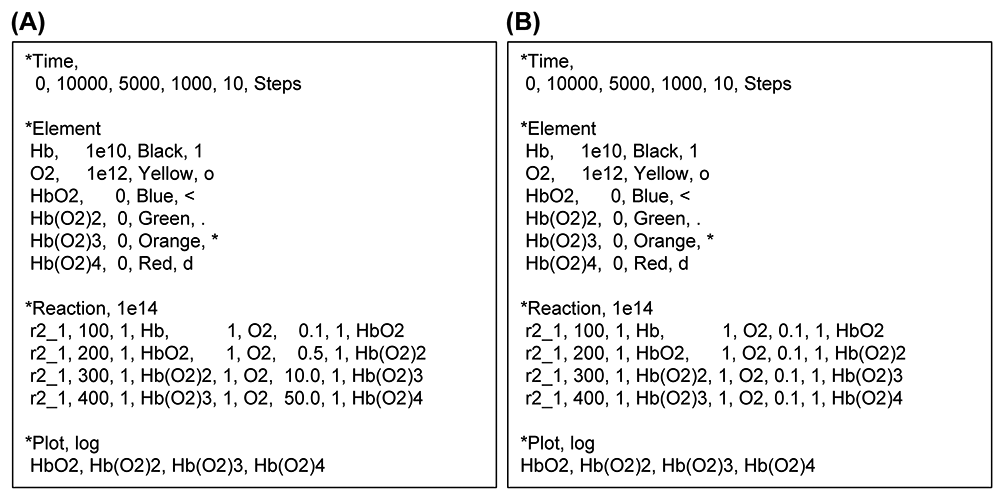


**Supplementary Figure S2.** Input text of the Monod–Wyman–Changeux allosteric model. (A) Rate constants increase with O_2_ binding. (B) Rate constants are equal.

Hb = hemoglobin, O_2_ = oxygen molecules, HbO_2_ = hemoglobin with one oxygen molecule, Hb(O_2_)_2_ = hemoglobin with two oxygen molecules, and so on.


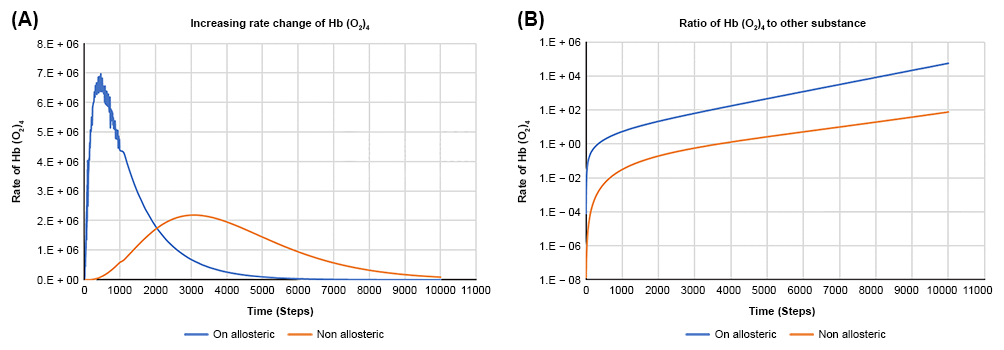


**Supplementary Figure S3.** Results of the Monod–Wyman–Changeux allosteric model.

“On allosteric” means results calculated from Figure S2A and “Non allosteric” is results from Figure S2B.

(**A**) Increasing rate change of Hb(O_2_)_4_ in Figure 5. (**B**) Ratio of Hb(O_2_)_4_ to other substance; HbO_2_, Hb(O_2_)_2_, and Hb(O_2_)_3_ in Figure 5.


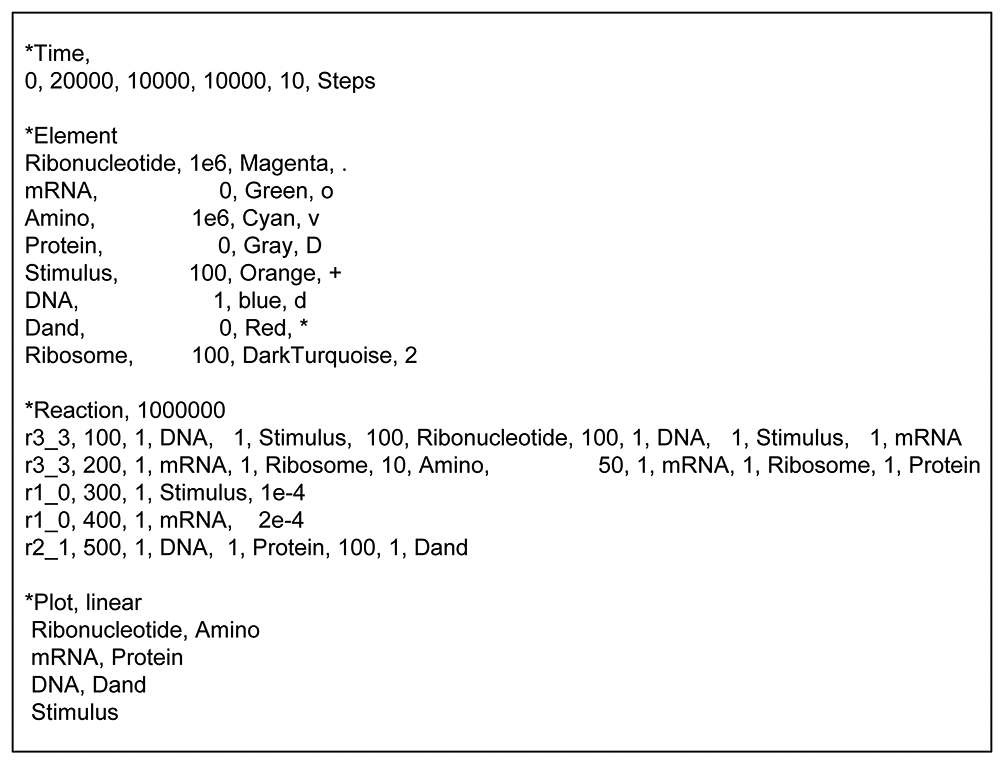


**Supplementary Figure S4**. Text input file for the feedback loop.


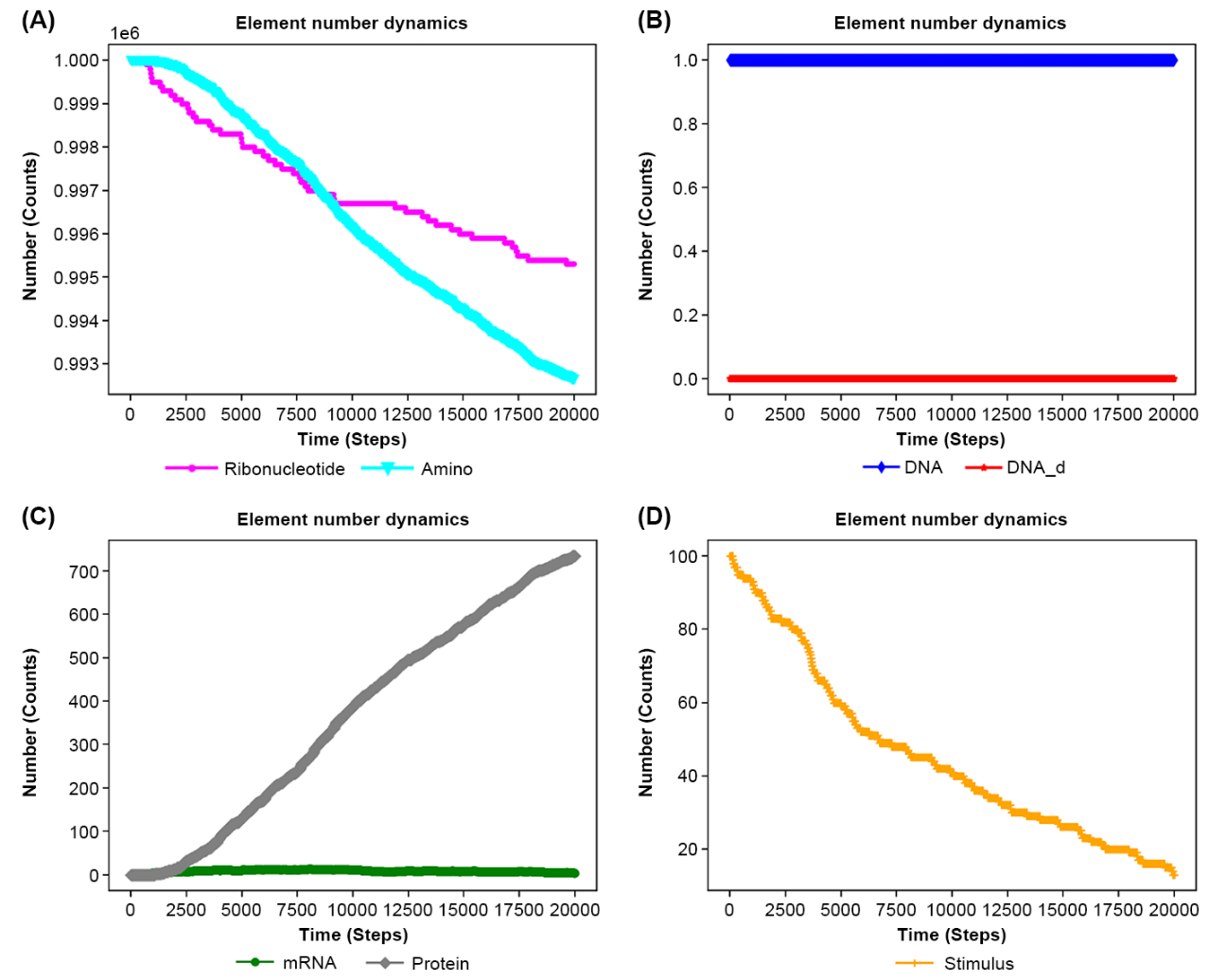


**Supplementary Figure S5.** Results of the feedback system using the input file in Supplementary Figure S4 without the reaction “r2_1_500.” (**A**) Ribonucleotide and Amino. (**B**) DNA and DNA_d. (**C**) mRNA and Protein. (**D**) Stimulus.





**Supplementary Figure S6.** Input file of the feed-forward system. *Element statements are changed and *ElementInOut in Supplementary Table S3 are added.





**Supplementary Figure S7.** Input file of the feed-forward system in Figure 8. Sx and Sy are added at the same time, t = 10000.


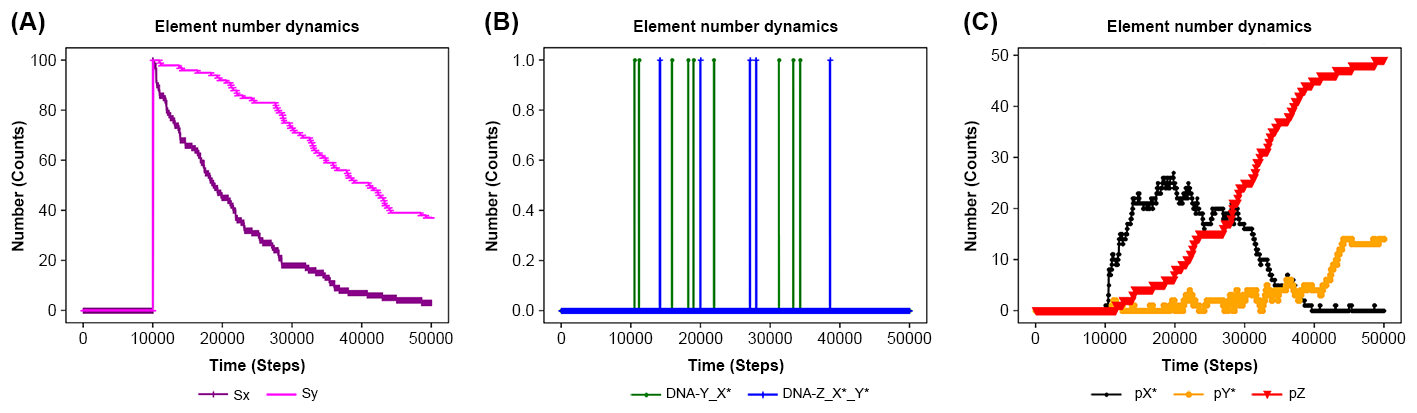


**Supplementary Figure S8.** Feed-forward system of Supplementary Figure S7 until time = 50000 steps. Sx and Sy are added at 5000 steps and 10000 steps, respectively. **(A)** Sx and Sy. **(B)** DNA-Y_X* and DNA-Z_X*_Y*. **(C)** pX*, pY*, and pZ.

**
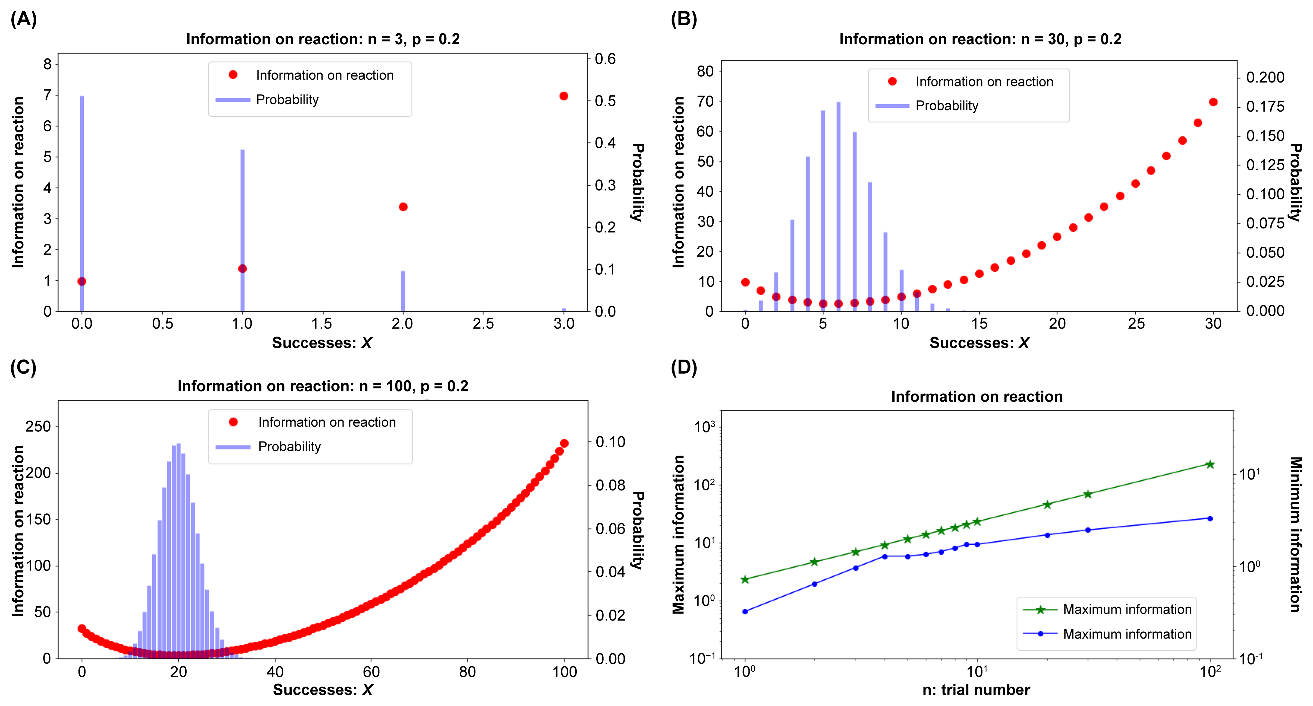
**

**Supplementary Figure S9.** Information on the reaction in Equation (19). **(A)** n = 3, p = 0.2. (**B**) n = 30, p = 0.2. (**C**) n = 300, p = 0.2. **(D)** Maximum and minimum information on reaction with p = 0.2.


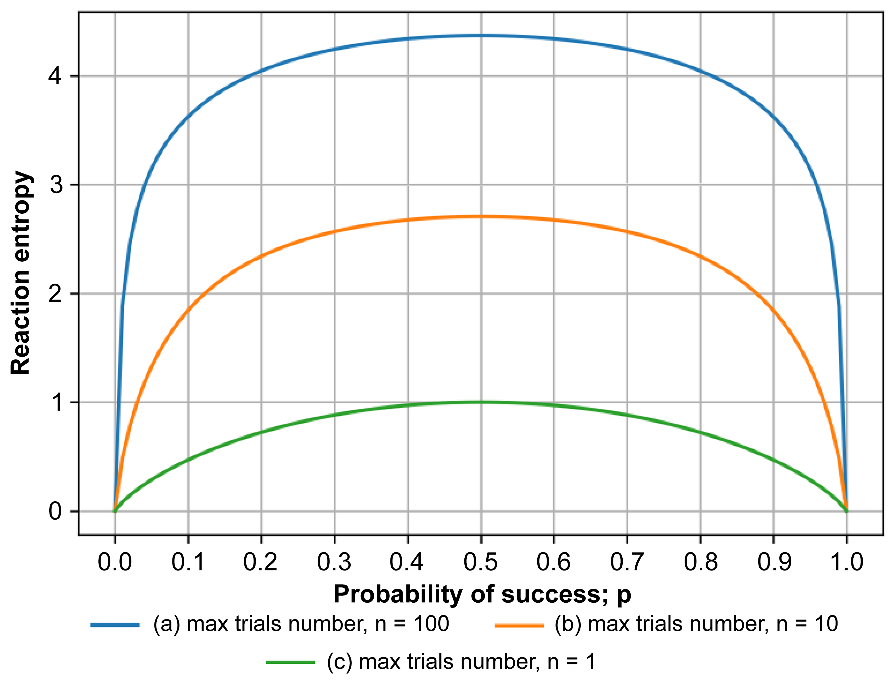


**Supplementary Figure S10.** Reaction entropy as a function of p at each of the three n values.
